# Draft Genome Sequence of *Thermomicrobium* sp. Strain 4228-Ro, a Thermophilic Bacterium Isolated from a Kamchatka Hot Spring

**DOI:** 10.1128/mra.01221-22

**Published:** 2023-02-22

**Authors:** Olga A. Podosokorskaya, Alexandra A. Klyukina, Tatiana V. Panova, Vladimir A. Rodin, Maria I. Zvereva, Adolf S. Tulenkov, Alexander V. Lebedinsky, Ilya V. Kublanov, Alexander G. Elcheninov

**Affiliations:** a Winogradsky Institute of Microbiology, Federal Research Center of Biotechnology, Russian Academy of Sciences, Moscow, Russia; b Faculty of Chemistry, Lomonosov Moscow State University, Moscow, Russia; c Center for educational programs in bioinformatics, Moscow Institute of Physics and Technology, Dolgoprudniy, Russia; d Faculty of Biology, Lomonosov Moscow State University, Moscow, Russia; Wellesley College

## Abstract

The genome of *Thermomicrobium* sp. strain 4228-Ro, an aerobic thermophilic bacterium isolated from a Kamchatka hot spring, was sequenced and analyzed. The genome assembly comprises 13 contigs with a total length of 3,068,448 bp. Genome analysis revealed the pathway of aerobic utilization of sugars, which was corroborated by growth experiments.

## ANNOUNCEMENT

Asediment sample was collected from a hot spring located in the Uzon Caldera, Kamchatka, Russia (temperature [T], 69°C; pH 6.2; N54 29.940 E159 59.529) in July 2021. Enrichment culture 4228 was obtained by adding the sediments (10%, vol/vol) to a 15-mL tube filled with 5 mL of aerobic modified Widdel medium (1) with pH 5.3, supplemented with glucose (1 g · L^−1^), ampicillin (100 μg · mL^−1^), and polymyxin B (10 μg · mL^−1^). After 17 days of incubation at 60°C, the dominating microorganisms were isolated using the dilution to extinction technique on the same medium without antibiotics. This antibiotic-free medium was used for strain 4228-Ro cultivation-based experiments, which were conducted in duplicate. Growth rate and cell yield were controlled by phase-contrast light microscopy.

For genomic sequencing, strain 4228-Ro was cultured under optimal conditions (60°C and pH 6.5) with glucose (1 g · L^−1^) for 4 days. For Illumina sequencing, genomic DNA of the strain was isolated using a DNeasy PowerLyzer microbial kit (Qiagen), and sequencing libraries were prepared using a HyperPlus kit (Kapa Biosystems) according to the manufacturer’s instructions. Paired-end sequencing (2 × 100 bp) was performed using an Illumina NovaSeq 6000 instrument. For Oxford Nanopore sequencing, the DNA was isolated with the Monarch genomic DNA purification kit (New England BioLabs). Nanopore sequencing was performed on a MinION instrument with the SQK-LSK109 protocol and R9.4.1 flow cell (Oxford Nanopore Technologies) and resulted in a total of 440,639,965 bases with *N*_50_/*N*_90_ values of 4,859/1,422 bp. Base calling was done using Guppy v.5.0.17 (2) with cutting Q at <7 for quality control. Unless otherwise stated, default parameters were used for all software. The initial genome assembly was performed with Flye v.2.9 (3) using Nanopore reads. A total of 20,862,292 raw Illumina reads with an average length of 101 bp were filtered in CLC Genomics Workbench v.10 (Qiagen) using Trim tool (quality limit = 0.03, maximum ambiguous nucleotides = 2, minimum length = 100). The Nanopore assembly was corrected in Pilon v.1.24 (4) (7 rounds of polishing) using 17,547,458 filtered Illumina reads. The resulting genome assembly statistics was inferred with QUAST v.5.0.2 (5). Completeness and contamination were measured using CheckM v.1.2.1 (6) with the bacteria-specific marker set. Genome annotation was performed using NCBI Prokaryotic Genome Annotation Pipeline v.6.3 (7).

The final assembly of the strain 4228-Ro genome comprises 13 contigs with a total length of 3,068,448 bp, an *N*_50_ value of 2,046,163 bp, and a G+C content of 65.03%. It includes 3 circular (1 chromosome, 1 megaplasmid, and 1 plasmid with sizes of 2,046,163, 907,128, and 37,846 bp, respectively) and 10 linear contigs, which are presumably the fragments of unassembled plasmid(s). The estimated completeness and contamination of the assembly were 98.28% and 0%, respectively. A total of 2,812 open reading frames (ORFs) were predicted, including 2,719 protein-coding, 6 rRNA, 50 tRNA, and 3 noncoding RNA (ncRNA) genes and 34 pseudogenes. Next, 16S rRNA gene sequence BLASTn (v.2.13.3, nonredundant nucleotide database) revealed that strain 4228-Ro belonged to the *Thermomicrobium* genus (*Chloroflexota*), which currently (8) includes two validly published species, namely, Thermomicrobium carboxidum KI3^T^ and Thermomicrobium roseum DSM 5159^T^. The genome encoded a semiphosphorylative Entner-Doudoroff pathway (9), a complete citrate cycle, and a cytochrome *c* oxidase, altogether determining heterotrophic aerobic growth on sugars. All genes considered indispensable for aerobic carbon monoxide oxidation (coxMSLDEFG [10]) were present in the genome, implying the capability of strain 4228-Ro of CO-trophy, which has been shown for *T*. *carboxidum* KI3^T^.

### Data availability.

The whole-genome sequence was deposited in GenBank under the accession number JAPFQM000000000. The BioProject, BioSample, and two SRA accession numbers are PRJNA899013, SAMN31636293, SRR22264076, and SRR22264077, respectively.
